# New Frontier in Advanced Dentistry: CBCT, Intraoral Scanner, Sensors, and Artificial Intelligence in Dentistry

**DOI:** 10.3390/s22082942

**Published:** 2022-04-12

**Authors:** Seong-Hun Kim, Ki Beom Kim, HyeRan Choo

**Affiliations:** 1 Department of Orthodontics, Graduate School of Dentistry, Kyung Hee University, Seoul 02447, Korea; 2 Department of Orthodontics, Center for Advanced Dental Education, Saint Louis University, Saint Louis, MO 63104, USA; kibeom.kim@health.slu.edu; 3 Department of Plastic and Reconstructive Surgery, Lucile Packard Children’s Hospital, Stanford University School of Medicine, Palo Alto, CA 94304, USA; hchoo@stanford.edu

The advancement of science and technology has brought innovation in the dental field. Both diagnosis and treatment have been dramatically changed by the evolution of imaging technology. Digital dentistry, which has become a big topic, is greatly correlated with the improvement of imaging technology. For example, cone beam computed tomography (CBCT) enables 3D skeletal dental analysis for treatment plans in orthodontic, prosthesis, periodontal, and orthognathic surgery. While the radiation dose is a fraction of medical spiral CT, it provides accurate enough information and further permits soft tissue and airway evaluation. The anatomical information and treatment outcome evaluation using CBCT allow for establishing accurate, safe, and innovative treatment guidelines. Intraoral/extraoral/model scanning allows the 3D simulation of dental treatment, and with 3D printers using these scanned data, we can fabricate therapeutic devices such as T-scans, electromyography, joint vibration analysis, and jaw--tracking devices to aid in dynamic analysis. The AI approach to 2D or 3D imaging technology is fundamentally changing the protocol of diagnosis in dentistry. 

This Special Issue delivers original research on many different aspects: studies on the application of CBCT for studying jaw growth, anatomic evaluation, and assessment of the CBCT-based 3D model’s accuracy; studies on the application of various force measurement sensors for bite force, disclusion time reduction therapy, and force distribution evaluation of dentition during implementation of innovative multilayer mandibular advancement devices for Obstructive Sleep Apnea treatment; and studies on non-radiation dose quantitative light-induced fluorescence (QLF) for early periodontal disease detection. This Special Issue also covers updated research about pathologic lesion detection with low-radiation-dose CBCT and computer-aided design/computer-aided manufacturing technology in terms of patient and occupational safety in the COVD-19 period. 

First, Chae JM et al.’s study evaluated midpalatal bone density (BD) by using CBCT according to gender, age, and vertical and horizontal skeletal patterns and found the higher BD in female group and Class II malocclusion group and a high reliability of age on BD increase [1]. This information is helpful for clinicians to understand our patients’ anatomies and potential hurdles in successful treatment. Yi L et al.’s team’s two consecutive longitudinal growth studies of the maxillomandibular complex in untreated children using CBCT showed us quite interesting findings in the three-dimensional growth direction and pattern [2,3]. Especially in the transverse dimension, when comparing maxillary dentoalveolar changes with that of the mandible, greater increases were noticed in the maxilla, which might be explained by the presence of sutural growth in the maxilla [2]. 

Three-dimensional models from CBCT images can be computed for the CAD/CAM-based fabrication of dental restorations or orthodontic devices. Cho MH et al. evaluated the 3D model accuracy of high-resolution micro-CT in terms of different cone beam angles and suggested that the model errors are smaller in the combined half-scan image reconstruction when the cone-beam angle is as large as 10 degrees [4]. Park JH et al.’s intraoral scanner study showed that the proper application of digital indirect bracket bonding system (IDBS) should be performed considering the errors, and resin-based fabrication might not be essential in ensuring high-accuracy IDBS [5]. 

Contrary to the CBCT and intraoral scanner technology, quantitative light-induced fluorescence (QLF) technology mainly focuses on the early detection of pathologic lesions of the teeth surface. Oh SH et al.’s study broadens the scope of QLF from dental caries or cracks to periodontal disease detection for the first time. They evaluated periodontal risk factors with oral health habits and fluorescent plaque index using QLF images to evaluate their effect on the degree of radiographic bone loss. The results of this study suggest that the clinical use of QLF enables plaque detection by non-invasive procedures and can aid in a more objective estimation for oral hygiene status [6].

Three research articles in this Special Issue focus on the clinical accuracy of occlusal force and dental pressure sensing devices. Thumati P et al.’s Disclusion Time Reduction (DTR) study at five Dental Colleges, using intraoral sensors and muscular electrodes, showed statistically significant improvement in various chronic muscular myofascial pain dysfunction symptom patients by immediate complete Anterior Guidance Development Coronoplasty compared to the control group [7]. Gao J et al.’s study of the newly developed portable biosensor using the sandwich technique showed that the mechanical stress-measuring device made by medical and industrial cross has a good application prospect for the measurement of bite force during function [8]. A new type of flexiforce sensor was used in Ahn HW et al.’s mandibular advancement device (MAD) study, which is a commonly used treatment modality for patients with mild-to-moderate obstructive sleep apnea. They analyzed the force distribution on the entire dentition according to the materials and design of the MADs. In addition, the core-reinforced multilayered MAD can reduce the force delivered to the dentition more effectively than the conventional single- or double-layer devices in their study [9]. 

Another topic investigated in this Special Issue is a microbial infection of sensing devices due to COVID-19 and the radiation hazard of the CBCT apparatus. Barenghi L et al. updated the evidence reported in their previous review on the advantages and limitations of computer-aided design/computer-aided manufacturing technology in the promotion of dental business, as well as to guarantee patient and occupational safety in the current COVID-19 pandemic [10]. Husain AAH et al.’s ex vivo CBCT study compared the low-dose mode to a standard-dose imaging for detecting pre-prepared cystic lesions in pig jaw models. Low-dose protocols provided confidential diagnostic evaluation with an improved benefit–risk ratio according to the ALALA principle and could become a promising alternative as a primary diagnostic tool, as well as for radiological follow-up in the treatment of cystic lesions [11].

In conclusion, we debated on the status of CBCT, intraoral scanners, force-measuring sensors, and healthcare issues in contemporary dentistry. We would like to suggest a correct direction of optical sensors and force sensors in this Special Issue because the comprehension of recent advances in sensing devices of dentistry would lead to appropriate applications of these sensors and successful strategies to improve treatment outcomes to better serve patients in the future digital 4D dentistry. 

## Data Availability

Not applicable.
